# Action of dexmedetomidine on the substantia gelatinosa neurons of the rat spinal cord

**DOI:** 10.1111/j.1460-9568.2008.06260.x

**Published:** 2008-06

**Authors:** Hideaki Ishii, Tatsuro Kohno, Tomohiro Yamakura, Miho Ikoma, Hiroshi Baba

**Affiliations:** Division of Anesthesiology, Niigata University Graduate School of Medical and Dental Sciences1-757 Asahimachi, Niigata 951-8510, Japan

**Keywords:** α_2_-adrenoceptor, analgesia, dexmedetomidine, dorsal horn, substantia gelatinosa, whole-cell patch-clamp

## Abstract

Dexmedetomidine is a highly specific, potent and selective α_2_-adrenoceptor agonist. Although intrathecal and epidural administration of dexmedetomidine has been found to produce analgesia, whether this analgesia results from an effect on spinal cord substantia gelatinosa (SG) neurons remains unclear. Here, we investigated the effects of dexmedetomidine on postsynaptic transmission in SG neurons of rat spinal cord slices using the whole-cell patch-clamp technique. In 92% of the SG neurons examined (*n*= 84), bath-applied dexmedetomidine induced outward currents at −70 mV in a concentration-dependent manner, with the value of effective concentration producing a half-maximal response (0.62 μm). The outward currents induced by dexmedetomidine were suppressed by the α_2_-adrenoceptor antagonist yohimbine, but not by prazosin, an α_1_-, α_2B_- and α_2C_-adrenoceptor antagonist. Moreover, the dexmedetomidine-induced currents were partially suppressed by the α_2C_-adrenoceptor antagonist JP-1302, while simultaneous application of JP-1302 and the α_2A_-adrenoceptor antagonist BRL44408 abolished the current completely. The action of dexmedetomidine was mimicked by the α_2A_-adrenoceptor agonist oxymetazoline. Plots of the current–voltage relationship revealed a reversal potential at around −86 mV. Dexmedetomidine-induced currents were blocked by the addition of GDP-β-S [guanosine-5′-*O*-(2-thiodiphosphate)] or Cs^+^ to the pipette solution. These findings suggest that dexmedetomidine hyperpolarizes the membrane potentials of SG neurons by G-protein-mediated activation of K^+^ channels through α_2A_- and α_2C_-adrenoceptors. This action of dexmedetomidine might contribute, at least in part, to its antinociceptive action in the spinal cord.

## Introduction

α_2_-Adrenoceptor agonists mediate a number of physiological phenomena such as antinociception (Yaksh, 1985). They produce an antinociceptive effect by action on the locus ceruleus (Guo *et al.*, 1996) and spinal cord (Yaksh, 1985; Takano & Yaksh, 1991). Clonidine was the first α_2_-adrenoceptor agonist to be introduced clinically. It has analgesic properties when given epidurally or intrathecally in humans. Epidural clonidine was found to be effective in reducing both postoperative pain (Anzai & Nishikawa, 1995) and intractable neuropathic cancer pain (Eisenach *et al.*, 1995). Intrathecal clonidine also decreased postoperative pain (Filos *et al.*, 1994). Dexmedetomidine is another α_2_-adrenoceptor agonist. Intravenous administration of dexmedetomidine has been used clinically for sedation in the intensive care unit (Venn & Grounds, 2001). Meanwhile, several lines of evidence indicate that administration of dexmedetomidine produces spinal analgesia as efficiently as clonidine. Behavioral studies in rats have demonstrated an inhibition of nociceptive responses by intrathecally (Fisher *et al.*, 1991) and epidurally (Asano *et al.*, 2000) administered dexmedetomidine. α_2_-Adrenoceptor agonists are also used in as adjuncts to general anesthesia. The effect of oral clonidine premedication can reduce volatile anesthetic (Ghignone *et al.*, 1987) and opioid (Ghignone *et al.*, 1986) requirements in the perioperative period. Intravenous administration of dexmedetomidine similarly diminishes the volatile anesthetic (Aho *et al.*, 1992) and opioid (But *et al.*, 2006) requirements.

Although both clonidine and dexmedetomidine are α_2_-adrenoceptor agonists, several differences exist between the two. First, the potency of dexmedetomidine is much greater than that of clonidine at maximum efficacy. Intraperitoneal administration of clonidine decreased minimum alveolar concentration, which produces immobility in 50% of subjects exposed to a noxious stimulus, for halothane about 40% in rats, whereas minimum alveolar concentration was decreased about 90% by dexmedetomidine (Maze *et al.*, 1987; Segal *et al.*, 1988). A second difference lies in the relative α_2_/α_1_ selectivity ratio. The α_2_/α_1_-adrenoceptor selectivity ratio of clonidine is 220 : 1 (Virtanen *et al.*, 1988). Accordingly, high doses of clonidine may induce cardiovascular side-effects via the α_1_-adrenoceptor (Virtanen *et al.*, 1988). The α_2_-/α_1_-adrenoceptor selectivity ratio of dexmedetomidine is 1620 : 1 (Virtanen *et al.*, 1988). As dexmedetomidine is a highly selective α_2_-adrenoceptor agonist (Savola & Virtanen, 1991), dexmedetomidine causes fewer side-effects mediated by α_1_-adrenoceptor activation than clonidine.

Neurons of the superficial dorsal horn, especially lamina II (substantia gelatinosa: SG), are thought to play an important role in modulating nociceptive transmission, because they preferentially receive thin myelinated Aδ- and unmyelinated C-primary afferent fibers, both of which carry nociceptive information from the periphery (Kumazawa & Perl, 1978; Yoshimura & Jessell, 1989). Moreover, binding and immunohistochemical studies show that the highest density of α_2_-adrenoceptors is in the superficial layers of the spinal dorsal horn (Roudet *et al.*, 1994; Stone *et al.*, 1998). However, whether dexmedetomidine exerts its effect on SG neurons remains unclear. In the present study, we used whole-cell patch-clamp recording to clarify whether dexmedetomidine has postsynaptic effects on SG neurons and to confirm the involvement of α_2_-adrenoceptors.

## Materials and methods

This study was approved by the Animal Research Committee of Niigata University Graduate School of Medical and Dental Sciences in Niigata, Japan. All efforts were made to minimize the number of animals used.

### Spinal cord slice preparation

Male Wistar rats (4–8 weeks old) were anesthetized with urethane (1.5 g/kg, i.p.). The lumbosacral segment of the spinal cord was removed as described previously (Yoshimura & Nishi, 1993; Kohno *et al.*, 2003) and placed in a preoxygenated ice-cold (1–3°C) sucrose-substituted Krebs solution containing (in mm): KCl 6.4, MgSO_4_ 4.1, NaHCO_3_ 26, NaH_2_PO_4_ 1.3, glucose 10 and sucrose 252, bubbled with 95% O_2_ and 5% CO_2_ (Wang *et al.*, 1999). Transverse spinal cord slices (500 μm) were cut on a vibrating microslicer. The slices were placed on a nylon mesh in a recording chamber, and then perfused at a rate of 10 ml/min with normal Krebs solution containing (in mM): NaCl 117, KCl 3.6, CaCl_2_ 2.5, MgCl_2_ 1.2, Na_2_HPO_4_ 1.2, NaHCO_3_ 25, glucose 11, at 36 ± 1°C for at least 1 h prior to recordings.

### Electrophysiological recordings

The lamina II was clearly discernible as a relatively translucent band across the dorsal horn under a dissecting microscope with transmitted illumination. Blind whole-cell patch-clamp recordings were made from SG neurons in voltage clamp mode using patch-pipette electrodes with a resistance of 5–10 MΩ (Yoshimura & Nishi, 1993; Kohno *et al.*, 2003). Two pipette solutions were used. The first solution contained (in mm): potassium gluconate 135, KCl 5, CaCl_2_ 0.5, MgCl_2_ 2, EGTA 5, HEPES 5 and ATP-Mg 5 (pH 7.2). Guanosine-5′-*O*-(2-thiodiphosphate) (GDP-β-S, 2 mm) was used as a GTP-binding protein blocker when necessary. The second solution contained (in mm): Cs_2_SO_4_ 110, tetraethylammonium (TEA) 5, CaCl_2_ 0.5, MgCl_2_ 2, EGTA 5, HEPES 5 and ATP-Mg 5 (pH 7.2). Cs and TEA were used as K^+^ channel blockers. Membrane currents were amplified using an Axopatch 200B amplifier (Molecular Devices). Signals were filtered at 2 kHz and digitized at 5 kHz. Data were collected and analysed using pClamp9.0 software (Molecular Devices).

### Application of drugs

Drugs were applied by superfusion without alteration of the perfusion rate or temperature. The drugs used were dexmedetomidine (provided by Abbott Japan), prazosin, yohimbine, tetrodotoxin (TTX) (Wako, Japan), oxymetazoline, GDP-β-S, acridin-9-yl-[4-(4-methylpiperazin-1-yl)-phenyl]amine (JP-1302) (Sigma-Aldrich, USA), 2-[(4,5-dihydro-1*H*-imidazol-2-yl)methyl]-2,3-dihydro-1-methyl-1*H*-isoindole maleate (BRL44408) (Tocris Bioscience, USA).

### Data analysis

Numerical data are expressed as means ± SEM. Statistical significance was assessed as *P*< 0.05 using the paired Student's *t*-test. In all cases, *n* refers to the number of neurons studied. Relative peak amplitude was calculated as an amplitude of dexmedetomidine-induced current in the presence of the drugs, divided by that of currents produced by dexmedetomidine alone. The continuous curve for the concentration–response relationship of dexmedetomidine was drawn according to the following Hill equation: *y*= 1.3/[1 + (EC_50_/*x*)^*b*^], where *x* is the dexmedetomidine concentration and *b* is the Hill coefficient.

## Results

### Dexmedetomidine induces currents in dorsal horn neurons

**Fig. 1:**
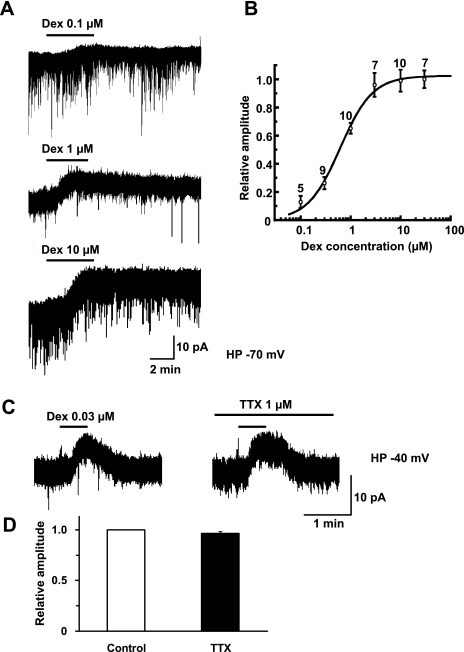
Dexmedetomidine induces an outward current in a concentration-dependent manner in SG neurons. (A) Outward currents induced by dexmedetomidine (0.1, 1 and 10 μm). Duration of drug superfusion is shown by horizontal bars above chart recordings. Holding potential = −70 mV. (B) Relative peak amplitude was calculated as an amplitude of the dexmedetomidine (0.1–30 μm)-induced current divided by that of the current produced by dexmedetomidine (30 μm). The continuous curve was drawn according to the Hill plot with an EC_50_ value of 0.62 μm (95% confidence interval, 0.51–0.77 μm) and a Hill coefficient of 1.34. The number next to each point denotes the number of neurons examined. Holding potential = −70 mV. (C) Outward currents elicited by dexmedetomidine (0.03 μm) in the absence and presence of TTX (1 μm). These currents were obtained from the same neuron (*n*= 5). Holding potential = −40 mV. (D) Relative peak amplitude was calculated as an amplitude of dexmedetomidine-induced current in the presence of TTX (5.3 ± 1.3 pA) divided by that of currents produced by dexmedetomidine (5.6 ± 1.4 pA) without TTX.

The resting membrane potentials in the SG neurons were −65.8 ± 0.8 mV (*n*= 17). In 92% of the SG neurons examined (*n*= 84), bath-applied dexmedetomidine induced outward currents at −70 mV. As shown in Fig. 1A, outward currents exhibited a clear dose-dependency on dexmedetomidine perfused at the surface of the spinal cord. The onset of the responses became slow and recovery was delayed with increasing concentrations of dexmedetomidine. Fig. 1B shows a concentration–response curve of the dexmedetomidine-induced outward currents. Analysis of the curve gave 0.62 μm as the effective concentration producing a half-maximal response (EC_50_) with a Hill coefficient of 1.34. A higher concentration of dexmedetomidine produced a long duration at a holding potential of −70 mV (Fig. 1A) and it was difficult to be applied repeatedly. Consequently, we used a low concentration of dexmedetomidine (0.03 μm) in order to reproduce responses when it was repeatedly applied. In addition, the dexmedetomidine (0.03 μm)-induced outward currents could not be observed at a holding potential of −70 mV, as shown in Fig. 1B. Therefore, we recorded the currents at a holding potential of −40 mV (Fig. 1C and D). To confirm that the dexmedetomidine-induced currents were postsynaptic effects, we examined the currents in the presence of TTX to remove any possible influence of α_2_-adrenoceptors on presynaptic terminals. We compared peak amplitude elicited by dexmedetomidine (0.03 μm) in the absence and presence of TTX (1 μm). TTX had no significant effect on the amplitude of dexmedetomidine (Fig. 1Cand D, 96.2 ± 1.3% of control, *P*= 0.91, *n*= 5).

### Pharmacological analysis of dexmedetomidine-induced responses

**Fig. 2:**
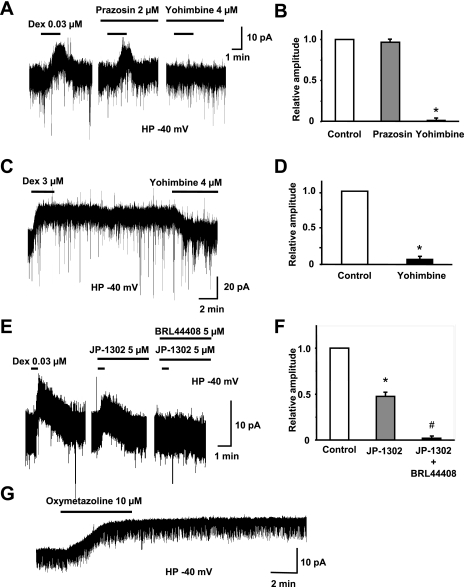
Dexmedetomidine-induced currents are mediated by α_2_- (α_2A_- and α_2C_-) but not by α_1_-adrenoceptors. (A) Prazosin (2 μm), an α_1_-adrenoceptor antagonist, did not affect the amplitude of the outward current induced by dexmedetomidine (0.03 μm), whereas the currents were suppressed by application of yohimbine (4 μm), an α_2_-adrenoceptor antagonist. These currents were obtained from the same neuron (*n*= 6). Holding potential = −40 mV. (B) Relative peak amplitude was calculated as an amplitude of dexmedetomidine-induced current in the presence of prazosin (8.7 ± 2.2 pA) or yohimbine (0.1 ± 0.1 pA) divided by that of currents produced by dexmedetomidine (9.3 ± 2.6 pA) alone. *P< 0.05, control vs. yohimbine. Holding potential = −40 mV. (C) Dexmedetomidine (3 μm) produced an outward current of long duration. A persistent outward current after washout of dexmedetomidine was completely reduced in amplitude by yohimbine (4 μm). Holding potential = −40 mV. (D) The dexmedetomidine-induced outward currents in the presence of yohimbine were significantly smaller than control (*P< 0.05, *n*= 10). (E) Application of JP-1302 (5 μm) partially blocked the dexmedetomidine (0.03 μm)-induced current, and simultaneous application of JP-1302 (5 μm) and BRL44408 (5 μm) abolished the current completely. These currents were obtained from the same neuron (*n*= 6). Holding potential = −40 mV. (F) Relative peak amplitude was calculated as an amplitude of dexmedetomidine-induced current in the presence of JP-1302 (3.3 ± 0.3 pA) or JP-1302 plus BRL44408 (0.1 ± 0.1 pA) divided by that of currents produced by dexmedetomidine (6.9 ± 0.6 pA) alone. *P< 0.05, control vs. JP-1302. ^#^*P*< 0.05, control vs. JP-1302 plus BRL44408. (G) Dexmedetomidine-induced outward current was mimicked by the α_2A_-adrenoceptor agonist oxymetazoline (10 μm) on SG neurons. Holding potential = −40 mV.

We examined the effects on dexmedetomidine-induced responses of the antagonists prazosin (2 μm) and yohimbine (4 μm), as compared with those of previous studies (prazosin 0.5–2 μm, yohimbine 1–4 μm) (Baba *et al.*, 2000; Kawasaki *et al.*, 2003; Sonohata *et al.*, 2004). The dexmedetomidine-induced outward currents were attenuated by the application of the α_2_-adrenoceptor antagonist yohimbine (4 μm), while the α_1_-adrenoceptor antagonist prazosin (2 μm) had no significant effect (Fig. 2A). Figure 2B shows a summary of the suppressive effects of each adrenergic antagonist. We next examined the effects on dexmedetomidine-induced responses using a lower concentration of prazosin (0.2 μm) and yohimbine (0.4 μm). However, these antagonists did not have any effect on the dexmedetomidine-induced outward currents (prazosin, 93.3 ± 4.1% of control, *P*= 0.71; yohimbine, 91.0 ± 7.4% of control, *P*= 0.88, *n*= 5). These results suggested that the dexmedetomidine-induced outward current was mediated by α_2_-adrenoceptors.

Application of a high concentration of dexmedetomidine (3 μm) produced an outward current of long duration. Superfusing yohimbine (4 μm) alone accelerated the recovery of outward currents to baseline level (Fig. 2Cand D), confirming that the dexmedetomidine-induced current was mediated by α_2_-adrenoceptors. Furthermore, to examine the involvement of α_2A_- and α_2C_-adrenoceptors on the dexmedetomidine-induced current, we used α_2A_- and α_2C_-adrenoceptor subtype-preferring antagonists (Fig. 2E andF). Application of the α_2C_-adrenoceptor antagonist JP-1302 (5 μm) partially suppressed the dexmedetomidine (0.03 μm)-induced current. In addition, it was markedly abolished when dexmedetomidine was again applied in the presence of JP-1302 (5 μm) and the α_2A_-adrenoceptor antagonist BRL44408 (5 μm). Relative peak amplitude was reduced to 3.3 ± 0.3 pA in the presence of JP-1302 and to 0.1 ± 0.1 pA in the presence of JP-1302 plus BRL44408 (dexmedetomidine alone, 6.9 ± 0.6 pA, Fig. 2F). We next used oxymatazoline, an α_2A_-adrenoceptor agonist (Miyazaki *et al.*, 1998; Kawasaki *et al.*, 2003). In nine of ten neurons, oxymatazoline (10 μm) induced an outward current similar to that of dexmedetomidine (Fig. 2G) with a peak amplitude of 10.2 ± 1.0 pA (*n*= 9). This result suggested that α_2A_-adrenoceptors were present and activatable on SG neurons. These findings indicated that both α_2A_- and α_2C_-adrenoceptors were involved in the dexmedetomidine-induced current.

### Dexmedetomidine activates K^+^ channels through the activation of G-proteins on SG neurons

**Fig. 3:**
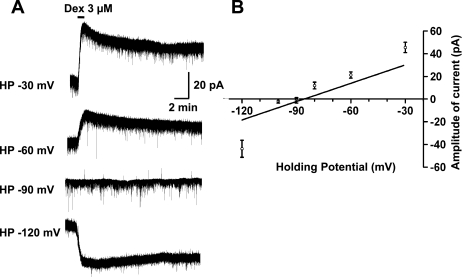
(A) Dexmedetomidine-induced currents show voltage dependency. (B) Relationship between holding potential and current amplitude. The regression equation was *y*= 0.901*x* + 77.1 [*R*^2^ = 0.92, *P*= 0.002, *y*= amplitude of current (pA), *x*= holding potential (mV)].

We next investigated what kinds of channels mediate the dexmedetomidine current. Figure 3A shows the dexmedetomidine (3 μm)-induced currents recorded at different holding potentials. Plots of the current–voltage relationship revealed a reversal potential at around −86 mV (Fig. 3B), which is slightly different from the equilibrium potential (−97 mV) of K^+^, as calculated from the Nernst equation using K^+^ concentrations ([K^+^]_o_, 3.6 mm; [K^+^]_i_, 140 mm) of the solutions. This slight difference might be considered to reflect a liquid junction potential (9–10 mV) existing between the Krebs and patch-pipette solutions (Yajiri *et al.*, 1997). Superfusing dexmedetomidine opens K^+^ channels on postsynaptic SG neurons, generating an outward current at holding potentials below −90 mV.

**Fig. 4:**
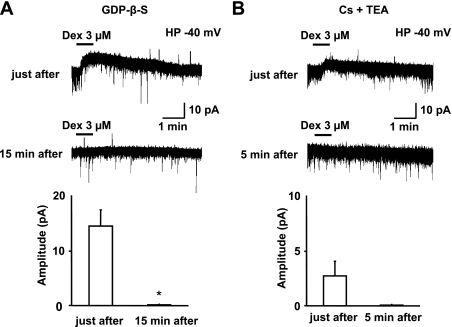
Inhibition of dexmedetomidine-induced current by GDP-β-S or Cs^+^/TEA. (A) The dexmedetomidine (3 μm)-induced outward current was examined with K-gluconate pipette solution containing GDP-β-S (2 mm). Dexmedetomidine produced an outward current just after establishing the whole-cell configuration, but this was markedly abolished when dexmedetomidine was again applied 15 min later (*P< 0.05, *n*= 5). Holding potential = −40 mV. (B) Dexmedetomidine (3 μm) induced an outward current just after establishing whole-cell recording with Cs^+^ and TEA-containing pipette. When dexmedetomidine was again applied 5 min later, the current was completely abolished (*n*= 7). Holding potential = −40 mV.

To determine whether G-proteins are responsible for the dexmedetomidine-induced current, GDP-β-S (2 mm), a non-hydrolysable analog of GDP that competitively inhibits G-proteins, was used in the pipette solution to prevent the postsynaptic activation of α_2_-adrenoceptors. When dexmedetomidine (3 μm) was applied shortly after establishing the whole-cell configuration, an outward current was observed (Fig. 4A). This outward current was completely abolished when dexmedetomidine was again applied 15 min later (Fig. 4A, *n*= 5). We next used the pipette solution containing Cs^+^ and TEA to inhibit the postsynaptic effect of K^+^ channels. Dexmedetomidine (3 μm)-induced outward current was recorded just after establishing the whole-cell configuration (Fig. 4B). However, this current was abolished after the second application of dexmedetomidine, which was performed more than 5 min later (Fig. 4B, *n*= 7). These findings suggested that the dexmedetomidine-induced outward current was mediated by K^+^ channels through the activation of G-proteins.

### Effects of dexmedetomidine on spontaneous excitatory postsynaptic currents

**Fig. 5:**
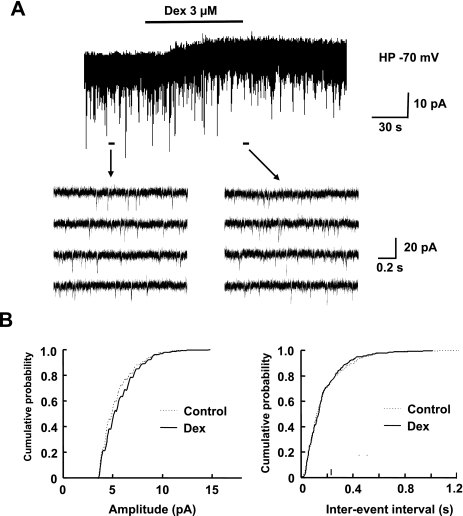
Dexmedetomidine dose not affect the frequency or amplitude of spontaneous EPSCs in SG neurons. (A) Continuous chart recording of sEPSCs before and during the application of dexmedetomidine (3 μm) (upper trace). Four consecutive traces of sEPSCs for a period indicated by a short bar below the chart recording are shown on an expanded time scale (lower traces). Holding potential = −70 mV. (B) Cumulative distributions of the amplitude (left) and the inter-event interval (right) of sEPSCs before (dashed line) and during the application of dexmedetomidine (continuous line). Dexmedetomidine had no significant effect on the amplitude (*P*> 0.05; Kolmogorov–Smirnov test) or the inter-event interval distribution (*P*> 0.05).

We next examined the effects of dexmedetomidine on spontaneous excitatory postsynaptic currents (sEPSCs) in SG neurons (Fig. 5A). Amplitude and inter-event interval distributions were not changed by dexmedetomidine (Fig. 5B; amplitude, *P*> 0.05, Kolmogorov–Smirnov test; inter-event interval, *P*> 0.05). These data indicate that dexmedetomidine does not affect glutamate release from presynaptic terminals.

## Discussion

Intrathecal dexmedetomidine is a highly potent antinociceptive agent in animals (Fisher *et al.*, 1991). Lipophilicity may affect the analgesic potency of intrathecally administered agents (Eisenach *et al.*, 1994). Highly lipophilic agents bind to spinal cord more efficiently than poorly lipophilic drugs. Dexmedetomidine is more lipophilic than clonidine (Savola *et al.*, 1986). Eisenach *et al.* (1994) showed that intrathecal administration of dexmedetomidine at a dose of 100 μg produced an antinociceptive effect that was first evident at 30 min and lasted for 90 min after the injection. As these authors demonstrated in a separate set of experiments in the same study, intrathecal administration of dexmedetomidine at this dose resulted in cerebrospinal fluid concentrations ranging from 300 to 3000 ng/ml (nearly 1–10 μm) during the period 30–90 min after injection. These results are consistent with the concentrations of dexmedetomidine that induced outward currents in the present study (Fig. 1B). Taken together, these data suggest that the cerebrospinal fluid concentration of dexmedetomidine required for antinociception is 1–10 μm.

Behavioral studies in rats have demonstrated that intrathecal administration of dexmedetomidine produces dose-dependent antinociception (Fisher *et al.*, 1991). Low doses of dexmedetomidine produced a transient period of antinociception. On the other hand, profound antinociception was prolonged by high doses of dexmedetomidine. For example, a transient current was evoked by a low concentration of dexmedetomidine (0.03 μm) in the present study (Fig. 2A). By contrast, a high concentration of dexmedetomidine (3 μm) produced a current with a long duration (Fig. 2C). The durations of dexmedetomidine (3 μm)-induced currents were longer than 30 min in most neurons without desensitization. The higher the concentration of dexmedetomidine, the longer the duration tended to be. The durations of dexmedetomidine-induced outward currents were much longer than those induced by noradrenaline (Sonohata *et al.*, 2004). This long duration may be caused by the high affinity of dexmedetomidine. A radioligand binding study showed that the binding affinity (pKi) value of dexmedetomidine was higher than that of noradrenaline for the α_2_-adrenoceptor (Jasper *et al.*, 1998). Alternatively, the long-term effect may be due to a slow rate of diffusion of dexmedetomidine out of the spinal cord slice (Koga *et al.*, 2005). This issue remains to be established.

As mentioned above, superficial dorsal horn neurons are important for processing nociceptive information from primary afferent fibers. Binding and immunohistochemical studies have suggested that the highest density of α_2_-adrenoceptors exist in the superficial layers of the spinal dorsal horn (Roudet *et al.*, 1994; Stone *et al.*, 1998). In addition, the binding sites of the α_2A_-adrenoceptors in the superficial dorsal horn were dramatically reduced in number after neonatal capsaicin treatment or dorsal rhizotomy, whereas α_2C_-adrenoceptors were not significantly reduced by either of these treatments. This finding suggests that α_2C_-adrenoceptors exist in postsynaptic sites (Stone *et al.*, 1998; Olave & Maxwell, 2002), and that α_2A_-adrenoceptors are located mainly in presynaptic sites (Stone *et al.*, 1998; Kawasaki *et al.*, 2003) of the dorsal horn. By contrast, few α_2B_-adrenoceptors were seen in the spinal dorsal horn by *in situ* hybridization studies of the distribution of α_2B_ mRNA (Nicholas *et al.*, 1993; Shi *et al.*, 1999). Furthermore, an α_2_-adrenoceptor agonist did not change spinal antinociception in α_2B_-adrenoceptor knockout mice, suggesting that α_2B_-adrenoceptors do not participate in the antinociceptive effect in the spinal cord (Fairbanks *et al.*, 2002). However, these immunohistochemical studies showed a dramatic, but incomplete, reduction in α_2A_-adrenoceptor immunoreactivity. This result raised the question of whether α_2A_-adrenoceptors were also located on postsynaptic SG neurons, in addition to α_2C_-adrenoceptors. We used prazosin as the α_1_-adrenoceptor antagonist, but prazosin acts not only as an α_1_-adrenoceptor, but also has some properties of α_2B_- and α_2C_-adrenoceptor antagonists (Bylund, 1988; MacDonald *et al.*, 1997). The binding inhibition coefficients (Ki) of prazosin were 2750, 108 and 98 nm for the α_2A_-, α_2B_- and α_2C_-adrenoceptor, respectively (Marjamaki *et al.*, 1993). Nevertheless, our study showed that dexmedetomidine-induced currents were not blocked by the α_2B_- and α_2C_-adrenoceptor antagonistic action of prazosin in SG neurons (Fig. 2A andB). Therefore, highly selective α_2A_- or α_2C_-adrenoceptor antagonists were superfused to investigate this question. BRL44408 was noted as a selective α_2A_-adrenoceptor antagonist having Ki of 4, 174 and 187 nm for the α_2A_-, α_2B_- and α_2C_-adrenoceptor, respectively (Bylund *et al.*, 1994). JP-1302 was recently described as a novel, highly selective α_2C_-adrenoceptor antagonist (Sallinen *et al.*, 2007). The Ki were 1500, 2200 and 16 nm for the α_2A_-, α_2B_- and α_2C_-adrenoceptor, respectively. Application of JP-1302 partially suppressed the dexmedetomidine-induced current, and simultaneous application of JP-1302 and BRL44408 abolished the amplitude of the current completely (Fig. 2EandF). Our findings clarify the involvement of both α_2A_- and α_2C_-adrenoceptors in the dexmedetomidine-induced current. Furthermore, we showed that the dexmedetomidine-induced current seen in postsynaptic SG neurons was mimicked by oxymetazoline, an α_2A_-adrenoceptor agonist (Fig. 2G). The Ki were 6, 3150 and 180 nm for the α_2A_-, α_2B_- and α_2C_-adrenoceptor, respectively (Marjamaki *et al.*, 1993). Taking these data together, it is likely that dexmedetomidine activates both α_2A_- and α_2C_-adrenoceptors on postsynaptic SG neurons and induces currents. As we did not examine the application of an α_2C_-adrenoceptor agonist, we could not conclude whether there were α_2C_-adrenoceptors in postsynaptic SG neurons. Further studies are required with highly selective α_2A_- or α_2C_-adrenoceptor agonists and antagonists.

We showed that activation of α_2_-adrenoceptors with dexmedetomidine resulted in outward currents that were mediated by activation of K^+^ channels through the activation of G-proteins (Figs 3 and4). These findings suggest that dexmedetomidine hyperpolarizes the membrane potentials of SG neurons by opening G-protein-coupled inwardly rectifying potassium (GIRK) channels. The GIRK1 and GIRK2 subunits are concentrated in lamina II of the mouse spinal cord (Marker *et al.*, 2005). As the antinociceptive effect of clonidine was reduced in GIRK2-knockout mice (Mitrovic *et al.*, 2003), it is possible that dexmedetomidine exerts its effects on GIRK channels similarly to clonidine.

We focussed on the postsynaptic effects of dexmedetomidine in SG neurons. However, α_2_-adrenoceptors are also localized on primary afferent terminals (Stone *et al.*, 1998). Noradrenaline acts on α_2_-adrenoceptors on postsynaptic (Sonohata *et al.*, 2004) and presynaptic (Kawasaki *et al.*, 2003) SG neurons, and produces an antinociceptive effect. Kawasaki *et al.* (2003) showed that noradrenaline, clonidine and oxymetazoline inhibited the peak amplitudes of monosynaptically evoked Aδ- and C-fiber EPSCs, while miniature EPSC (mEPSC) amplitude and frequency was unaffected by noradrenaline. Pan *et al.* (2002) reported that the peak amplitude of evoked EPSCs was attenuated by clonidine in the outer zone of SG. Furthermore, clonidine significantly decreased the frequency of mEPSCs. A discrepancy between this and the current study may be due to the fact that different SG neurons were tested; Pan *et al.* examined neurons in the outer layer of SG, while we investigated neurons located at the center of SG. The possibility cannot be ruled out that SG neurons exhibiting no effect of noradrenaline on mEPSCs (Kawasaki *et al.*, 2003) or no effect of dexmedetomidine on sEPSCs in the current study (where the blind patch-clamp technique was used) had located in the inner layer of SG because visually identified neurons in the inner layer of SG appeared to be without actions of clonidine on mEPSCs (Pan *et al.*, 2002). Li & Zhuo (2001) showed that clonidine inhibited glutamate-mediated evoked EPSCs. However, clonidine preferentially depressed polysynaptic but not monosynaptic Aδ-fiber-evoked field potentials in superficial spinal dorsal horn (Ruscheweyh & Sandkuhler, 2000). Moreover, α_2_-adrenoceptor agonists depressed the NMDA receptor-mediated excitatory postsynaptic potential on A- and C-primary afferent fibers (Faber *et al.*, 1998). Our results showed that dexmedetomidine did not affect glutamate release from presynaptic terminals. Whether dexmedetomidine exhibits a similar presynaptic action on these currents needs to be established.

In conclusion, the present study suggests that dexmedetomidine hyperpolarizes the membrane potentials of spinal dorsal horn neurons via G-protein-mediated activation of K^+^ channels through α_2_-adrenoceptors. This action of dexmedetomidine might contribute, at least in part, to its antinociceptive action in the spinal cord.

## Abbreviations

BRL44408: 2-[(4,5-dihydro-1*H*-imidazol-2-yl)methyl]-2,3-dihydro-1-methyl-1*H*-isoindole maleate
EPSCs: excitatory postsynaptic currents
GDP-β-S: guanosine-5′-*O*-(2-thiodiphosphate)
GIRK: G-protein-coupled inwardly rectifying potassium
JP-1302: acridin-9-yl-[4-(4-methylpiperazin-1-yl)-phenyl]amine
Ki: binding inhibition coefficient
pKi: binding affinity
SG: substantia gelatinosa
TEA: tetraethylammonium
TTX: tetrodotoxin

## References

[b1] Aho M, Erkola O, Kallio A, Scheinin H, Korttila K (1992). Dexmedetomidine infusion for maintenance of anesthesia in patients undergoing abdominal hysterectomy. Anesth. Analg..

[b2] Anzai Y, Nishikawa T (1995). Thoracic epidural clonidine and morphine for postoperative pain relief. Can. J. Anaesth..

[b3] Asano T, Dohi S, Ohta S, Shimonaka H, Iida H (2000). Antinociception by epidural and systemic alpha(2)-adrenoceptor agonists and their binding affinity in rat spinal cord and brain. Anesth. Analg..

[b4] Baba H, Shimoji K, Yoshimura M (2000). Norepinephrine facilitates inhibitory transmission in substantia gelatinosa of adult rat spinal cord (part 1): effects on axon terminals of GABAergic and glycinergic neurons. Anesthesiology.

[b5] But AK, Ozgul U, Erdil F, Gulhas N, Toprak HI, Durmus M, Ersoy MO (2006). The effects of pre-operative dexmedetomidine infusion on hemodynamics in patients with pulmonary hypertension undergoing mitral valve replacement surgery. Acta Anaesthesiol. Scand..

[b6] Bylund DB (1988). Subtypes of alpha 2-adrenoceptors: pharmacological and molecular biological evidence converge. Trends Pharmacol. Sci..

[b7] Bylund DB, Eikenberg DC, Hieble JP, Langer SZ, Lefkowitz RJ, Minneman KP, Molinoff PB, Ruffolo RR, Trendelenburg U (1994). International Union of Pharmacology nomenclature of adrenoceptors. Pharmacol. Rev..

[b8] Eisenach JC, Shafer SL, Bucklin BA, Jackson C, Kallio A (1994). Pharmacokinetics and pharmacodynamics of intraspinal dexmedetomidine in sheep. Anesthesiology.

[b9] Eisenach JC, DuPen S, Dubois M, Miguel R, Allin D (1995). Epidural clonidine analgesia for intractable cancer pain. The Epidural Clonidine Study Group. Pain.

[b10] Faber ES, Chambers JP, Evans RH (1998). Depression of NMDA receptor-mediated synaptic transmission by four alpha2 adrenoceptor agonists on the in vitro rat spinal cord preparation. Br. J. Pharmacol..

[b11] Fairbanks CA, Stone LS, Kitto KF, Nguyen HO, Posthumus IJ, Wilcox GL (2002). alpha(2C)-Adrenergic receptors mediate spinal analgesia and adrenergic-opioid synergy. J. Pharmacol. Exp. Ther..

[b12] Filos KS, Goudas LC, Patroni O, Polyzou V (1994). Hemodynamic and analgesic profile after intrathecal clonidine in humans. A dose-response study. Anesthesiology.

[b13] Fisher B, Zornow MH, Yaksh TL, Peterson BM (1991). Antinociceptive properties of intrathecal dexmedetomidine in rats. Eur. J. Pharmacol..

[b14] Ghignone M, Quintin L, Duke PC, Kehler CH, Calvillo O (1986). Effects of clonidine on narcotic requirements and hemodynamic response during induction of fentanyl anesthesia and endotracheal intubation. Anesthesiology.

[b15] Ghignone M, Calvillo O, Quintin L (1987). Anesthesia and hypertension: the effect of clonidine on perioperative hemodynamics and isoflurane requirements. Anesthesiology.

[b16] Guo TZ, Jiang JY, Buttermann AE, Maze M (1996). Dexmedetomidine injection into the locus ceruleus produces antinociception. Anesthesiology.

[b17] Jasper JR, Lesnick JD, Chang LK, Yamanishi SS, Chang TK, Hsu SA, Daunt DA, Bonhaus DW, Eglen RM (1998). Ligand efficacy and potency at recombinant alpha2 adrenergic receptors: agonist-mediated [35S]GTPgammaS binding. Biochem. Pharmacol..

[b18] Kawasaki Y, Kumamoto E, Furue H, Yoshimura M (2003). Alpha 2 adrenoceptor-mediated presynaptic inhibition of primary afferent glutamatergic transmission in rat substantia gelatinosa neurons. Anesthesiology.

[b19] Koga A, Fujita T, Totoki T, Kumamoto E (2005). Tramadol produces outward currents by activating mu-opioid receptors in adult rat substantia gelatinosa neurones. Br. J. Pharmacol..

[b20] Kohno T, Moore KA, Baba H, Woolf CJ (2003). Peripheral nerve injury alters excitatory synaptic transmission in lamina II of the rat dorsal horn. J. Physiol..

[b21] Kumazawa T, Perl ER (1978). Excitation of marginal and substantia gelatinosa neurons in the primate spinal cord: indications of their place in dorsal horn functional organization. J. Comp. Neurol..

[b22] Li P, Zhuo M (2001). Cholinergic, noradrenergic, and serotonergic inhibition of fast synaptic transmission in spinal lumbar dorsal horn of rat. Brain Res. Bull..

[b23] MacDonald E, Kobilka BK, Scheinin M (1997). Gene targeting – homing in on alpha 2-adrenoceptor-subtype function. Trends Pharmacol. Sci..

[b24] Marjamaki A, Luomala K, Ala-Uotila S, Scheinin M (1993). Use of recombinant human alpha 2-adrenoceptors to characterize subtype selectively of antagonist binding. Eur. J. Pharmacol..

[b25] Marker CL, Lujan R, Loh HH, Wickman K (2005). Spinal G-protein-gated potassium channels contribute in a dose-dependent manner to the analgesic effect of mu- and delta- but not kappa-opioids. J. Neurosci..

[b26] Maze M, Birch B, Vickery RG (1987). Clonidine reduces halothane MAC in rats. Anesthesiology.

[b27] Mitrovic I, Margeta-Mitrovic M, Bader S, Stoffel M, Jan LY, Basbaum AI (2003). Contribution of GIRK2-mediated postsynaptic signaling to opiate and alpha 2-adrenergic analgesia and analgesic sex differences. Proc. Natl Acad. Sci. U.S.A..

[b28] Miyazaki T, Kobayashi H, Tosaka T (1998). Presynaptic inhibition by noradrenaline of the EPSC evoked in neonatal rat sympathetic preganglionic neurons. Brain Res..

[b29] Nicholas AP, Pieribone V, Hokfelt T (1993). Distributions of mRNAs for alpha-2 adrenergic receptor subtypes in rat brain: an in situ hybridization study. J. Comp. Neurol..

[b30] Olave MJ, Maxwell DJ (2002). An investigation of neurones that possess the alpha 2C-adrenergic receptor in the rat dorsal horn. Neuroscience.

[b31] Pan YZ, Li DP, Pan HL (2002). Inhibition of glutamatergic synaptic input to spinal lamina II(o) neurons by presynaptic alpha(2)-adrenergic receptors. J. Neurophysiol..

[b32] Roudet C, Mouchet P, Feuerstein C, Savasta M (1994). Normal distribution of alpha 2-adrenoceptors in the rat spinal cord and its modification after noradrenergic denervation: a quantitative autoradiographic study. J. Neurosci. Res..

[b33] Ruscheweyh R, Sandkuhler J (2000). Differential actions of spinal analgesics on mono-versus polysynaptic Adelta-fibre-evoked field potentials in superficial spinal dorsal horn in vitro. Pain.

[b34] Sallinen J, Hoglund I, Engstrom M, Lehtimaki J, Virtanen R, Sirvio J, Wurster S, Savola JM, Haapalinna A (2007). Pharmacological characterization and CNS effects of a novel highly selective alpha2C-adrenoceptor antagonist JP-1302. Br. J. Pharmacol..

[b35] Savola JM, Virtanen R (1991). Central alpha 2-adrenoceptors are highly stereoselective for dexmedetomidine, the dextro enantiomer of medetomidine. Eur. J. Pharmacol..

[b36] Savola JM, Ruskoaho H, Puurunen J, Salonen JS, Karki NT (1986). Evidence for medetomidine as a selective and potent agonist at alpha 2-adrenoreceptors. J. Auton. Pharmacol..

[b37] Segal IS, Vickery RG, Walton JK, Doze VA, Maze M (1988). Dexmedetomidine diminishes halothane anesthetic requirements in rats through a postsynaptic alpha 2 adrenergic receptor. Anesthesiology.

[b38] Shi TJ, Winzer-Serhan U, Leslie F, Hokfelt T (1999). Distribution of alpha2-adrenoceptor mRNAs in the rat lumbar spinal cord in normal and axotomized rats. Neuroreport.

[b39] Sonohata M, Furue H, Katafuchi T, Yasaka T, Doi A, Kumamoto E, Yoshimura M (2004). Actions of noradrenaline on substantia gelatinosa neurones in the rat spinal cord revealed by in vivo patch recording. J. Physiol..

[b40] Stone LS, Broberger C, Vulchanova L, Wilcox GL, Hokfelt T, Riedl MS, Elde R (1998). Differential distribution of alpha2A and alpha2C adrenergic receptor immunoreactivity in the rat spinal cord. J. Neurosci..

[b41] Takano Y, Yaksh TL (1991). Relative efficacy of spinal alpha-2 agonists, dexmedetomidine, clonidine and ST-91, determined in vivo by using N-ethoxycarbonyl-2-ethoxy-1,2-dihydroquinoline, an irreversible antagonist. J. Pharmacol. Exp. Ther..

[b42] Venn RM, Grounds RM (2001). Comparison between dexmedetomidine and propofol for sedation in the intensive care unit: patient and clinician perceptions. Br. J. Anaesth..

[b43] Virtanen R, Savola JM, Saano V, Nyman L (1988). Characterization of the selectivity, specificity and potency of medetomidine as an alpha 2-adrenoceptor agonist. Eur. J. Pharmacol..

[b44] Wang MY, Rampil IJ, Kendig JJ (1999). Ethanol directly depresses AMPA and NMDA glutamate currents in spinal cord motor neurons independent of actions on GABAA or glycine receptors. J. Pharmacol. Exp. Ther..

[b45] Yajiri Y, Yoshimura M, Okamoto M, Takahashi H, Higashi H (1997). A novel slow excitatory postsynaptic current in substantia gelatinosa neurons of the rat spinal cord in vitro. Neuroscience.

[b46] Yaksh TL (1985). Pharmacology of spinal adrenergic systems which modulate spinal nociceptive processing. Pharmacol. Biochem. Behav..

[b47] Yoshimura M, Jessell TM (1989). Primary afferent-evoked synaptic responses and slow potential generation in rat substantia gelatinosa neurons in vitro. J. Neurophysiol..

[b48] Yoshimura M, Nishi S (1993). Blind patch-clamp recordings from substantia gelatinosa neurons in adult rat spinal cord slices: pharmacological properties of synaptic currents. Neuroscience.

